# Characteristics of residual lymph nodes after six months of antituberculous therapy in HIV-negative individuals with cervical tuberculous lymphadenitis

**DOI:** 10.1186/s12879-019-4507-0

**Published:** 2019-10-21

**Authors:** Hyeri Seok, Ji Hoon Jeon, Kyung Ho Oh, Hee Kyoung Choi, Won Suk Choi, Young Hen Lee, Hyung Suk Seo, Soon You Kwon, Dae Won Park

**Affiliations:** 1 Division of Infectious Diseases, Department of Medicine, Korea University Ansan Hospital, Korea University Medicine, 123 Jeukgeum-ro, Danwon-gu, Ansan, 15355 Republic of Korea; 2 Department of Radiology, Korea University Ansan Hospital, Korea University Medicine, Ansan, Republic of Korea; 3 Department of Otorhinolaryngology-Head and Neck Surgery, Korea University Ansan Hospital, Korea University Medicine, Ansan, Republic of Korea

**Keywords:** Residual lymph node, Cervical tuberculous lymphadenitis, Antituberculous treatment, Duration of treatment, Extrapulmonary tuberculousis

## Abstract

**Background:**

The therapeutic response of cervical tuberculous lymphadenitis (CTBL) may be delayed or paradoxical, with the frequent development of residual lymph nodes (LNs) during and after antituberculous treatment. We investigated the incidence of residual LNs and the clinical, radiological, microbiological, and pathologic responses of patients with CTBL after 6 months of antituberculous therapy.

**Methods:**

The medical records of HIV-negative adult patients with CTBL diagnosed between July 2009 and December 2017 were analyzed. After 6 months of first-line antituberculous treatment, computed tomography (CT) scans were conducted to evaluate for residual LNs. Fine-needle aspiration biopsy (FNAB) was carried out if a patient presented with residual LNs > 10 mm in diameter with central necrosis, peripheral rim enhancement, or perinodal inflammation on CT scan.

**Results:**

Residual LNs were detected in 35 of 157 patients who underwent follow-up CT scans and were more commonly observed in younger patients who completed the treatment (mean years ± standard deviation [SD]: 33 ± 13 vs. 44 ± 16, *p* < 0.001). The recurrence rate was approximately 5%, which was not significantly different in both groups. Among the 15 patients who underwent FNAB, 3 (30%) presented with granuloma, and 2 of 15 and 10 of 14 patients had positive AFB and TB PCR results, respectively. The TB culture results of 15 patients were negative.

**Conclusions:**

Residual LNs may still be observed after 6 months of antituberculous treatment. Although the radiologic and pathologic findings after treatment are still indicative of TB, not all residual LNs indicate recurrence or treatment failure. A six-month therapy may be sufficient for cervical tuberculous lymphadenitis.

## Background

Tuberculous lymphadenitis is one of the most common manifestations of extrapulmonary tuberculosis (TB) [1–4]. Although the total number of TB cases is declining in developed countries, the cases of extrapulmonary TB are not significantly decreasing, and such condition accounts for approximately 15–20% of all TB cases [1]. Approximately 30–50% of all extrapulmonary TB cases involve the peripheral LNs, of which cervical LN involvement is most common [2–5]. In Korea, 51.5 million individuals were newly diagnosed with TB in 2018 according to the declining trend. The proportion of extrapulmonary TB increased up to 21%, and tuberculous lymphadenitis accounted for 20% of extrapulmonary TB [6].

Although the duration of treatment for tuberculous lymphadenitis has been controversial, a six-month treatment is recommended for drug-susceptible organisms based on studies showing no significant differences in treatment failure or complication [7–9]. However, evidence supporting this recommendation for all cases of tuberculous lymphadenitis is limited because, unlike in pulmonary TB, there is no clear criteria for the assessment of extrapulmonary TB after treatment. The characteristics of extrapulmonary TB differ from those of pulmonary TB. First, the rates of drug susceptibility tests are relatively low because the rate of positive culture is low. Second, the response to antituberculous treatment is delayed, and paradoxical reactions occur more frequently in individuals with extrapulmonary TB than in those with pulmonary TB. Residual LNs may be observed in 12–30% of patients after antituberculous treatment [5]. Therefore, clinicians often encounter residual LNs after the six-month treatment, and there are controversies in relation to the interpretation of residual LNs. Some studies have recommended that an additional therapeutic approach is required due to the possibility of recurrence or resistance, and some have argued that prolonged treatment is not necessary because paradoxical reactions may occur [10–12]. Meanwhile, recent studies have shown that not all residual LNs indicate treatment failure. Additional treatment is recommended on the basis of microbiological testing results showing that LN is still found after short-term observation.

However, the evaluation of treatment failure in all residual LNs is challenging due to the limitations in terms of invasive tests and low diagnostic yield. Non-invasive modalities used for the evaluation of residual LNs after antituberculous chemotherapy are required. One study has indicated that computed tomography (CT) scan of the neck may be useful for the evaluation of treatment response [13]. A comparison of neck CT scan results before and after therapy for cervical tuberculous lymphadenitis (CTBL) has shown that the incidence of central necrosis, perinodal infiltration, and peripheral rim enhancement was significantly lower in the treatment success group than in the treatment failed group.

We described the clinical, radiological, microbiological and pathologic characteristics of residual LNs after six-month of antituberculous therapy in human immunodeficiency virus (HIV)-negative individuals with CTBL.

## Methods

### Study design and population

A prospective study was conducted to identify the clinical course and characteristics of CTBL. We enrolled adult patients older than 18 years who were diagnosed with CTBL between July 2009 and December 2017 at the Korea University Ansan Hospital, a 780-bed tertiary teaching hospital in Ansan, Republic of Korea. We excluded patients with HIV infection. All patients with CTBL were diagnosed via fine-needle aspiration biopsy (FNAB); the patients with CTBL who presented with cervical LN involvement only after completing the first-line antituberculous treatment were re-assessed. Such treatment comprised isoniazid (5 mg/kg), rifampin (10 mg/kg), ethambutol (25 mg/kg), and pyrazinamide (15–20 mg/kg) daily for the first 2 months, followed by isoniazid, rifampicin, and a reduced dose of ethambutol (15 mg/kg) for the next 4 months. Follow-up CT scans were performed within 1 month after six-month of antituberculous treatment. Re-FNAB was performed in patients with residual LN on follow-up CT scans if possible. The treatment of patients who maintained antituberculous drugs due to residual LNs was discontinued after confirming that *Mycobacterium tuberculosis* was not isolated from the culture. We compared the characteristics of the patients with and without residual LNs. The study protocol was approved by the institutional review board of Korea University Ansan Hospital (no. 2009AS0050). Written informed consent was obtained from each study participant.

### Definition

The diagnosis had been confirmed either by the detection of acid-fast bacilli (AFB) in smears from FNA or from biopsy and/or by positive mycobacterial culture of aspirates or biopsy and/or by histological evidence of TB. The histologic findings indicative of TB included necrotizing or caseating granuloma and necrosis without granuloma. When only cellular components were observed on cytological testing, TB was defined as the presence of histiocytes, multinucleated giant cells, and infiltrating lymphocytes [14]. The CT findings indicative of TB include central low density and peripheral rim enhancement with thick and irregular patterns [15]. The residual LN on CT scan after treatment was defined as LN > 10 mm in diameter with enhancement pattern of TB and central low attenuation with peripheral rim enhancement [13, 16]. Treatment failure was defined as residual LN that is > 10 mm in diameter and culture positivity after treatment. Recurrence was defined as reappearance of an LN or the appearance of a new tuberculous node after the completion of antituberculous therapy with a period of initial clinical remission, as previously described [9].

### Microbiology

Aspirates and digested fresh tissues were used for microscopic examination (Ziehl–Neelsen stain), culture, and TB PCR. For TB culture, samples were cultured on liquid and solid culture media. *M. tuberculosis* was identified using a commercial DNA probe (AccueProbe *Mycobacterium tuberculosis* Complex Culture Identification Test; Gen-Prove, San Diego, CA, the USA). A commercially available PCR test kit (COBAS® AMPLICORMTB; Roche Diagnostics, Branchburg, NJ, the USA) was used to assay fresh and fixed samples.

### Statistical analysis

To compare the two groups, Pearson χ^2^ tests and Fisher’s exact tests were used for categorical variables, and student’s *t*-test and Mann–Whitney U tests were utilized for continuous variables if appropriate. All statistical tests were two-tailed, and *p*-values ≤0.05 were considered statistically significant.

## Results

### Baseline clinical characteristics and outcomes of patients with CTBL

**Fig. 1:**
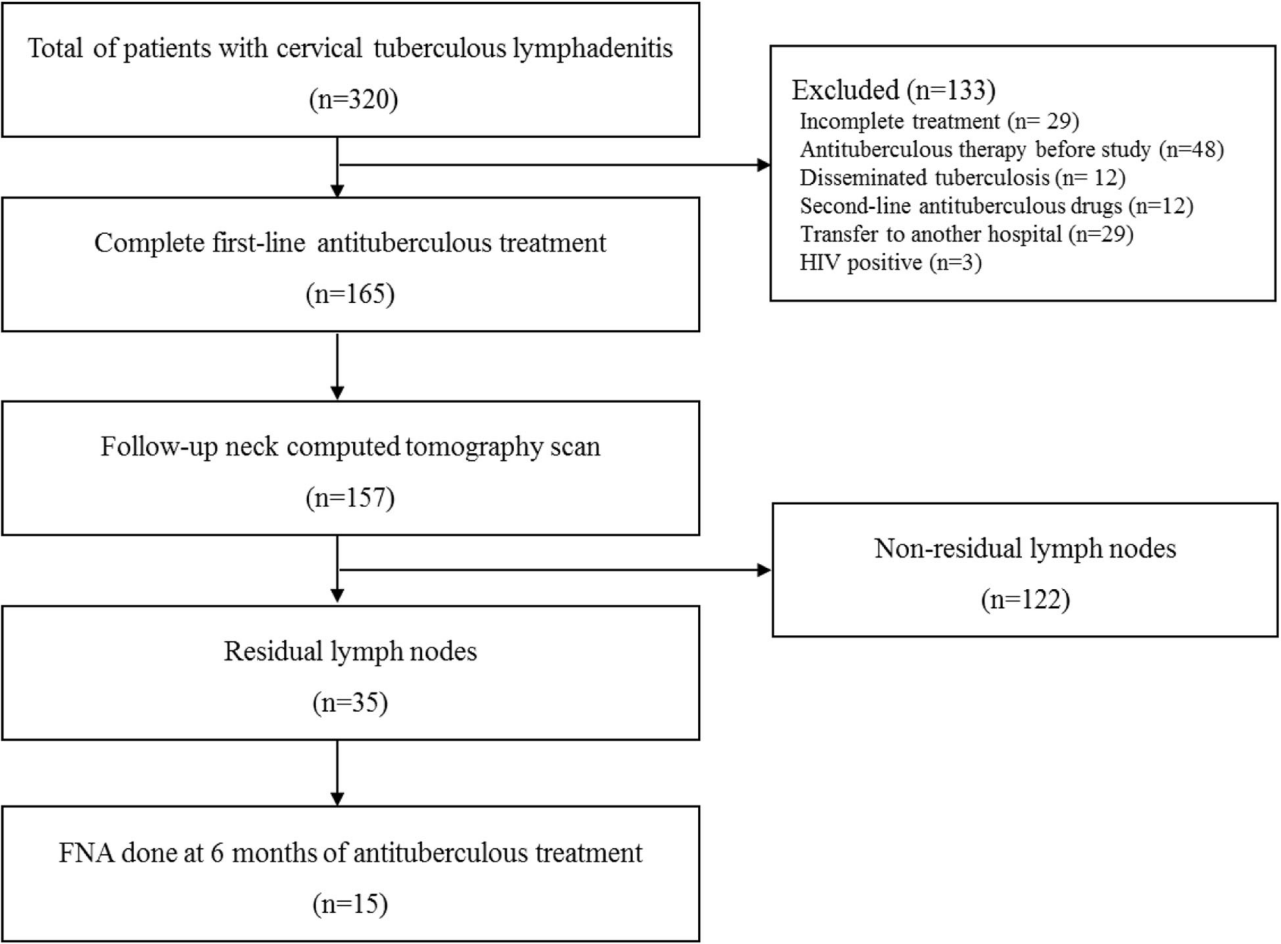
Flowchart of patients in this study

During the study period, 320 patients with CTBL were under antituberculous treatment (Fig. 1). One hundred thirty-three patients were excluded due to incomplete treatment, disseminated TB, resistance to first-line antituberculous drugs, or transfer to another hospital. One hundred sixty-five patients completed the first-line antituberculous treatment, and the baseline characteristics of the participants are presented in Table 1. Thirteen patients had a previous history of TB (tuberculous lymphadenitis, pulmonary TB, tuberculous pleurisy, and tuberculous peritonitis in the order of frequency). Nine of 13 patients have completed antituberculous treatment, and 130 patients did not have any underlying comorbidity. More than half of the patients presented with right cervical lymphadenopathy. At the time of initial diagnosis, the CT scan finding of 123 patients were indicative of TB. All 165 lymph nodes were larger than 1 cm. Hypodense lesions and rim enhancement were observed in 58 and 18 patients, respectively. Eighteen patients had central necrosis. Follow-up CT scans were carried out in 157 of 165 patients, and residual LNs after six-month treatment were observed in 35 (22.2%) patients. Thirty-two of 35 patients with residual LNs had one or more of the following findings on CT scan: central necrosis, perinodal infiltration, or peripheral rim enhancement. We observed paradoxical reactions during treatment in 38 (23.0%) patients. Four patients experienced spontaneous rupture and 12 patients underwent aspiration or drainage procedures just prior to rupture. Twelve patients were treated with NSAID and five patients received steroid treatment. The average treatment duration was 8 months with a median of 6 months. The median follow-up period was 658 days.

**Table 1:** Baseline clinical characteristics of patients with cervical tuberculous lymphadenitis

	All patients (*n* = 165)
Gender (male)	40 (24.2%)
Age (years, mean ± SD)	42.5 ± 16.1
Previous history of TB	13 (7.9%)
Comorbidity	35 (21.2%)
Cardiovascular diseases	25 (15.2%)
Diabetes mellitus	11 (6.7%)
Chronic renal diseases	7 (4.2%)
Hematologic malignancy	4 (2.4%)
Duration of lymphadenopathy (months, mean ± SD)	8.2 ± 29.8
Location of cervical lymph node	
Bilateral	14 (8.5%)
Left side	59 (35.8%)
Right side	92 (55.8%)
Largest size of the lymph node (mm, mean ± SD)	33.1 ± 24.5
Results of the initial diagnostic work-up	
Compatible with TB on CT scan, n/N^a^	123/143 (86.0%)
AFB positive, n/N^a^	20/96 (20.8%)
TB culture positive, n/N^a^	36/80 (45.0%)
TB PCR positive, n/N^a^	121/158 (76.6%)
Histologic findings indicative of TB	136/164 (82.9%)
Paradoxical reaction during treatment	38 (23.0%)
CT scan after 6 months of antituberculous therapy	157 (95.2%)
Residual lymph node^b^	35 (22.3%)
Duration of antituberculous therapy (months, mean ± SD)	7.9 ± 4.4

^a^No. of patients tested

^b^Residual lymph node was defined as a lymph node larger than 10 mm in diameter with central necrosis, peripheral rim enhancement, or perinodal infiltration on computed tomography scan

### Clinical comparisons of patients with and without residual LN

We compared the clinical characteristics of patients who presented with residual LNs on follow-up CT scans after 6 months of antituberculous treatment and those who did not (Table 2). Patients who presented with residual LNs after antituberculous treatment were significantly younger than those without (33 ± 13 versus 44 ± 16 years, *P* value < 0.001). No significant differences were observed between the two groups in terms of history of TB, comorbidities, size and location of LNs, and paradoxical reactions during the treatment. The duration of antituberculous treatment was extended in 34 patients among 35 patients who presented with residual LNs. The duration of treatment was significantly longer in the residual LN group than in the non-residual group (mean: 11.8 ± 7.5 vs. 7.0 ± 2.4 months; median: 9 (8–12) months vs 6 (6) months; *P* < 0.001). The recurrence rate after treatment was approximately 5%. However, the result was not significantly different between the two groups. Two patients in the residual LN group experienced recurrence after 2 months and 32 months after the discontinuation of treatment, respectively. In other patients, there were no clinical signs suggesting recurrence or treatment failure, including increased size of LNs.

**Table 2:** Comparisons of the clinical characteristics and treatment duration in patients with and without residual lymph nodes on follow-up CT scan after 6 months of antituberculous therapy

	Non-residual LN (*n* = 122)	Residual LN (*n* = 35)	*P*-value
Gender (male)	27 (22.1%)	11 (31.4%)	0.269
Age (years, mean ± SD)	43.96 ± 15.74	33.37 ± 13.36	< 0.001
Previous history of TB	9 (7.4%)	4 (11.4%)	0.488
Comorbidity	28 (23.0%)	3 (8.6%)	0.089
Duration of lymphadenopathy (months, mean ± SD)	9.36 ± 34.20	4.39 ± 10.95	0.187
Location of cervical lymph node			0.769
Bilateral	11 (9.0%)	3 (8.6%)	
Left side	46 (37.7%)	11 (31.4%)	
Right side	65 (53.3%)	21 (60.0%)	
Largest size of the lymph node (mm, mean ± SD)	32.77 ± 23.31	36.77 ± 29.71	0.466
Results of the initial diagnostic work-up, n/N^a^			
Compatible with TB on CT scan, n/N^a^	89/108 (82.4%)	26/27 (96.3%)	0.077
AFB positive, n/N^a^	17/77 (22.1%)	2/15 (13.3%)	0.728
TB culture positive, n/N^a^	27/63 (42.9%)	7/14 (50.0%)	0.768
TB PCR positive, n/N^a^	90/117 (76.9%)	26/33 (78.8%)	1.00
Histologic findings indicative of TB, n/N^a^	100/121 (82.6%)	30/35 (85.7%)	0.800
Paradoxical reaction during treatment	32/122 (26.2%)	5/35 (14.3%)	0.178
Duration of anti-tuberculous therapy (months)			
Mean ± SD	7.03 ± 2.40	11.82 ± 7.46	< 0.001
Median, IQR	6 (6–6)	9 (8–12)	< 0.001
Recurrence after antituberculous therapy	5/122 (4.1%)	2/35 (5.7%)	0.653

*Abbreviation*: *SD* Standard deviation, *TB* Tuberculosis, *CT* Computed tomography, *AFB* Acid-fast bacilli, *IQR* Interquartile range

^a^No. of patients tested

### FNAB results of residual LNs after the completion of antituberculous treatment

FNAB were performed in 15 of 35 patients with residual LNs on follow-up CT scans after six-month antituberculous treatment. The clinical characteristics of 15 patients are presented in Table 3. The most common pathologic findings were lymphoid cells with or without necrosis in 5 patients. Chronic granulomatous inflammation was observed in three patients. AFB stain and TB-PCRresults were positive in 2 (13.3%) of 15 and 10 (71.4%) of 14 patients, respectively. TB culture results were all negative in 15 patients. The treatment duration was shorter in the FNAB group than in the non-FNAB group (median months [interquartile range]: 8 [8–15] vs 10.5 [9–12], *P* < 0.001). None of the patients who underwent FNAB experienced treatment failure.

**Table 3:** Comparison of FNAB findings before and after 6 months of antituberculous treatment

	Initial evaluation	Follow-up evaluation after 6 months of treatment
	(*n* = 165)	(*n* = 15)
Mycobacterial findings		
TB-PCR	121/158 (76.6%)	10/14 (71.4%)
AFB stain	20/96 (20.8%)	2/15 (13.3%)
TB culture	36/80 (45.0%)	0/15 (0%)
Pathologic findings		
Granulomatous inflammation	136/164 (82.9%)	3/13 (23.0%)

*Abbreviation*: *FNAB* Fine-needle aspiration/biopsy, *TB* Tuberculosis, *PCR* Polymerase chain reaction, *AFB* Acid-fast bacilli

## Discussion

In this study, cervical tuberculous lymphadenopathy remains in almost a quarter of the patients on CT scans after 6 months of treatment, and none experienced treatment failure.

Lymphadenopathy usually disappears in 30–40% of patients after 3 months of antituberculous chemotherapy and in 80% after 6 months of treatment. However, LN that is > 5 mm in diameter may last for a long period of time in 20% of patients [9]. Although all residual LNs do not have an unfavorable outcome, defined as treatment failure or relapse [9], treatment might be prolonged or re-started in real world because unfavorable outcomes were not only on bacteriological or histological examination but also according to clinical findings. In contrast to smear-positive pulmonary TB, bacteriological treatment cannot be confirmed because it is challenging to obtain specimens from tuberculous lymphadenitis. In hospitals with poor healthcare facilities, decision-making in terms of treatment usually depends on clinical judgement when post-treatment lymphadenopathy occurs. In Korea where the prevalence of TB and the rate of drug-resistance is high, antituberculous treatment may be prolonged to 9–12 months when residual LNs remain [11].

In the absence of a consensus regarding the interpretation of the post-treatment residual LNs, this study revealed the characteristics of residual LNs based on pathologic findings. Biopsy or culture of residual nodes showed granuloma formation and negative culture results with or without positive AFB stains and/or TB PCR previously described as post-treatment paradoxical response [17, 18]. These features are consistent with hypersensitivity to the antigen of *M. tuberculosis*, which may be poorly cleared from the disease site even after prolonged therapy [1, 18]. Notably, not all residual LNs indicate treatment failure.

The risk factors of residual LNs must be identified because residual LNs, not treatment failure, can occur in some patients. However, the risk factors are not fully elucidated to date. Prior studies have indicated the following risk factors: younger age, male gender, size ≥3 cm, and local tenderness [11, 12, 17]. In our study, younger age was significantly correlated to residual LNs, which is consistent with previous studies. Thus, residual LNs may appear at the end of the six-month antituberculous therapy in younger patients, and treatment failure must be confirmed through bacteriological examination after short-term observation rather than prolonged treatment or drug changes.

Non-invasive methods, such as CT or fluorodeoxyglucose (FDG)-positron emission tomography (PET), and the identification of clinical risk factors may be helpful in evaluating the treatment response for tuberculous lymphadenitis [13, 16, 19]. Previous studies have shown that central necrosis, perinodal infiltration, and peripheral rim enhancement on CT scans were more frequently observed in patients with treatment failure [13]. When a higher SUVmax value from FDG is added to this CT scan findings, distinguishing the treatment responder and non-responder was more helpful [16]. Therefore, the size of the LN at the end of antituberculous treatment was not associated with treatment failure.

In the treatment period, the guidelines recommends six-month treatment for tuberculous lymphadenitis caused by drug-susceptible organisms [1, 20, 21]. The six-month recommendation is supported by studies showing that no difference was observed between 6 and 9 months of treatment in terms of cure rates (89–94%) [8, 22] or relapse rates (3%) [9]. The Joint Tuberculosis Committee of the British Thoracic Society has stated that follow-up is not required after a successful treatment. However, patients should be re-referred if symptoms recur due to the limited number of relapse cases [9, 23]. In this study, the duration of antituberculous treatment with residual LNs was shorter in the group whose microbiological characteristics were identified through FNAB. Considering the microbiological characteristics of residual LNs, this study may be supporting the six-month therapy in the preexisting guideline.

During the follow-up period with a median of 658 days, no differences were observed in terms of recurrence between the groups with and without residual LNs. However, recurrences are diagnosed without microbiological findings in patients with recurrence, which may reflect post-treatment paradoxical response rather than recurrence [17]. The gold standard for confirming recurrence is mycobacterial culture. However, culture alone cannot diagnose recurrence because of low sensitivity. Prior studies have already pointed out these problems [9, 17]. In one study, among patients with recurrence, three were diagnosed with recurrence according to mycobacterial culture results and based on histological findings, indicating that clinical judgement is still critical in identifying recurrence [9]. The recurrence period is usually within 12 months after the end of treatment. However, recurrence may occur after 1 year. Therefore, further investigation must be conducted as it remains unclear how long the required follow-up period is for residual LNs after antituberculous treatment.

This study presents the clinical, radiologic, and pathologic findings at the end of six-month treatment for tuberculous lymphadenitis. The limitations of this study are as follows: FNAB was not performed in all patients with residual LNs, and some of the specimens were inappropriate. Most patients with residual LNs were treated with antituberculous drugs for more than 6 months, and CT scans were not obtained in further follow up course. The variables correlated to the recurrence of TB among the residual LN group must be identified next. Further follow-up studies with a higher number of patients must be conducted for statistical significance.

## Conclusion

The presence of residual LNs after 6 months of antituberculous treatment in patients with CTBL does not necessarily indicate recurrence or treatment failure, even though radiologic and pathologic findings are consistent with TB. In younger patients, residual LNs may remain after 6 months of treatment, and re-evaluation may be considered after short-term observation. This study indicated that the prolonged treatment period, which has been commonly observed among patients with residual LNs, should be reconsidered and that a six-month antituberculous therapy may be sufficient.

## Data Availability

All data used in analysis of this manuscript is freely available by contacting the corresponding author.

## Abbreviations

AFB: Acid-fast bacilli
CT: Computed tomography
CTBL: Cervical tuberculous lymphadenitis
FDG-PET: Fluorodeoxyglucose-positron emission tomography
FNAB: Fine needle aspiration biopsy
HIV: Human immunodeficiency virus
LN: Lymph node
PCR: Polymerase chain reaction
SD: Standard deviation
TB: Tuberculosis
