# Correction to: Characteristics of residual lymph nodes after six months of antituberculous therapy in HIV-negative individuals with cervical tuberculous lymphadenitis

**DOI:** 10.1186/s12879-019-4640-9

**Published:** 2019-11-26

**Authors:** Hyeri Seok, Ji Hoon Jeon, Kyung Ho Oh, Hee Kyoung Choi, Won Suk Choi, Young Hen Lee, Hyung Suk Seo, Soon Young Kwon, Dae Won Park

**Affiliations:** 1 Division of Infectious Diseases, Department of Medicine, Korea University Ansan Hospital, Korea University Medicine, 123 Jeukgeum-ro, Danwon-gu, Ansan, 15355 Republic of Korea; 2 Department of Radiology, Korea University Ansan Hospital, Korea University Medicine, Ansan, Republic of Korea; 3 Department of Otorhinolaryngology-Head and Neck Surgery, Korea University Ansan Hospital, Korea University Medicine, Ansan, Republic of Korea

**Correction to: BMC Infect Dis (2019) 19:867**

**https://doi.org/10.1186/s12879-019-4507-0**

After publication of the original article [1], we were notified that an author’s name has been incorrectly spelled. Soon You Kwon’s correct full name is Soon Young Kwon.

The original article has been corrected.
